# Basic medical sciences and understanding medicine: Insights from medical students

**DOI:** 10.1371/journal.pone.0325749

**Published:** 2025-06-09

**Authors:** Dima Alhomsi, Mhd Khaled Tello, Dania Abdalsalam, Majd Barmo, Hamzeh Al Asadi, Hasan Najjar, Bayan Alsaid

**Affiliations:** 1 Faculty of Medicine, Damascus University, Syria; 2 Al-Mouassat University Hospital, Damascus, Syria; 3 Laboratory of Anatomy, Faculty of Medicine, Damascus University, Syria; University of Bremen: Universitat Bremen, GERMANY

## Abstract

**Introduction:**

Basic medical sciences form the cornerstone of any medical knowledge and are essential for understanding various diseases. This study aims to shed light on students’ viewpoints toward those sciences while delving into their motivations and hindrances and investigating individual subjects.

**Methods:**

This cross-sectional study was carried out at Damascus University in Syria, targeting final-year medical students. Data collection occurred on a designated clerkship day, during which students were invited to complete an anonymous, self-administered questionnaire. The questionnaire evaluated students’ overall perception and academic interest in studying basic medical sciences in pre-clinical years and their willingness to pursue these sciences as a prospective career.

**Results:**

The study surveyed 350 medical students, with 40% rating their interest as good/very good. The extensive content was perceived as a major hindrance by 76.9%. Anatomy was perceived as the easiest subject while pharmacology and biochemistry were considered the hardest. The majority acknowledged the value of basic sciences for further medical education (75.1%) and good physician (77.4%), but only 20.9% indicated their relevance for practical training. Furthermore, 68.6% of students were not interested in pursuing these sciences as a career, primarily due to their preference for clinical fields (55.8%) and concerns about low-income potential (41.3%).

**Conclusion:**

This study highlighted students’ positive views on Basic Medical Sciences, emphasizing their essential role in understanding disease pathophysiology for medical education and practice. Nonetheless, it pointed out a lack of practical training. The findings underscore the importance of decreasing content intensity and enhancing retention through practical approaches aligned with clinical practice.

## Introduction

Basic medical sciences refer to a group of sciences that focus on studying the human body’s formation and how its organs coordinately interact to maintain organic stability [1,2]. Basic medical sciences form the cornerstone of medical knowledge and are essential for understanding various diseases [3]. Many medical students rely on previously learned basic sciences to build their clinical knowledge [4].

Many studies have been conducted to explore students’ opinions regarding basic medical sciences and revealed varying findings. Students from higher classes felt that these sciences were less related to clinical practice [5]. Moreover, they wondered why they were taught subjects not quite relevant to the clinical practice and informally pointed out that they did not remember much from their first-year classes [6]. Many students in clinical years expressed that memorizing a lot of clinical information makes recalling basic medical information difficult [7].

Nevertheless, some research has highlighted positive perspectives. For example, a study conducted in India revealed that more benefits can be derived from these sciences by linking them with clinical practice [8]. Another study showed that they may play a role in clinical practice by helping students link diagnoses to their clinical manifestations. Additionally, it indicated that students with a good cognitive background in basic sciences tend to retain diagnostic-related information more than those without such a background [9].

Amidst the confusion in studies, a key issue in basic medical sciences is the strong emphasis on theoretical knowledge, which creates a gap between learning and practical application in clinical settings [10]. Recognizing this issue, countries like Nigeria have developed integrated curricula to enhance the learning experience and better connect knowledge to clinical practice [11].

Previous studies have often evaluated students’ perceptions of basic medical sciences without addressing specific subjects, and a definitive consensus among students remains lacking. Additionally, there is a notable absence of research in Syria exploring these perspectives. Gaining insights into students’ views on the relevance and challenges of basic medical sciences can provide valuable guidance for curriculum improvement.

This study aims to provide a comprehensive assessment of students’ perspectives on basic medical sciences, focusing on individual subjects. By identifying the factors that influence their interest or lack of interest in studying these sciences, as well as their reluctance to pursue careers in these fields, the findings can inform future curriculum adjustments. Strengthening the alignment between basic science education and clinical practice could create a more integrated and effective learning experience for medical students while raising awareness of the importance of these sciences.

## Methods

### Study design and setting

A cross-sectional study was conducted among final-year medical students at Damascus University, Syria in 2024.

### Participants

Participants authorized for inclusion in this study were final-year medical students who had completed three years of basic sciences and two years of clinical sciences, along with their respective clinical rotations. These students had studied all practical and theoretical aspects of medical subjects at Damascus University, passed all their exams, and enrolled to complete their final year, which consists solely of clinical rotations, at hospitals affiliated with Damascus University. These inclusion criteria ensured that participants had a thorough insight into basic subjects and their application during clinical years, enabling them to provide a comprehensive evaluation. Students who declined to participate or had incomplete or inaccurate data were excluded.

### The questionnaire

The questionnaire utilized in this study was meticulously designed to collect precise and reliable data on students’ perceptions of basic medical sciences. To ensure content validity, a comprehensive literature review of major databases was conducted to identify pertinent studies and validated questionnaires [2,12–14]. Relevant elements were adapted to align with the specific objectives of this study. Additionally, new items were developed to capture a more in-depth understanding of students’ perceptions of individual sciences, as well as their attitudes toward pursuing careers in these fields.

The questionnaire was drafted in English and then translated into Arabic using a forward-backward translation approach to ensure linguistic and conceptual equivalence. Bilingual researchers with backgrounds in medical education conducted the translations, and discrepancies were resolved by consensus to maintain the original meaning of the questions.

The final version of the questionnaire comprised four sections. The first section covered participants’ demographics (age, gender, and average college score). The second assessed academic interest in studying basic sciences during pre-clinical years, hindrances and motivations for studying them, and interest in various subjects. The third section evaluated the general perception of those sciences. Lastly, students’ willingness to pursue them as a prospective career was investigated.

A pilot study involving 40 medical students from the same population was conducted to ensure the questionnaire’s clarity. Participants provided feedback on items’ clarity, relevance, and wording, leading to minor linguistic adjustments for improved readability. No major amendments were necessary, indicating that the questionnaire was well-understood and suitable for the target population. This step ensured the questionnaire’s appropriateness for assessing students’ perceptions of basic medical sciences.

### Sampling

Formal enrollment records from Damascus University were reviewed to obtain a representative sample, estimating a population size of 1320 sixth-year students organized into 48 distinct groups to attend their mandatory clerkship rotations. Using the online sample size calculator Roasoft and considering a confidence interval of 95% and a marginal error of 5%, a minimum sample size of 298 was required.

Ensuring the generalizability of our findings, we adopted the aforementioned groups as our sampling framework. Utilizing the Simple Random Sampling technique, twelve groups were selected each consisting of approximately 30 students to exceed the required sample size. To minimize selection bias, all students within each chosen group were invited to complete an anonymous, paper-based, self-administered questionnaire on a designated clerkship day, affirming that the selected participants represent the broader student population. Recognizing potential self-reporting bias, participants were assured of anonymity and encouraged to provide honest responses without concern for evaluation. Data collection was conducted between 21/3/2024 and 21/4/2024.

After applying the exclusion criteria, a final sample size of 350 was achieved, yielding a response rate of 98%. To enhance data reliability and reduce potential data entry errors, responses were thoroughly proofread during entry, and incomplete or inconsistent data were excluded from the analysis.

### Ethical considerations

We conducted our study according to the Helsinki Declaration for research involving human subjects. Ethical approval was taken from the Biomedical Research Ethics Committee (BMREC) at Damascus University, Syria with ID number (MD-210224–191). Participation was voluntary, verbal consent was taken from each participant before distributing questionnaires with a question asking for written consent at the beginning of the questionnaire. The participant’s identity was kept anonymous.

### Data analysis

Descriptive statistics involved frequency and percentage for categorical variables, and mean and standard deviation for continuous variables. Spearman’s rank correlation coefficient was utilized to assess the correlation between students’ average grade and their interest in basic sciences. To analyze students’ perception, one sample t-test was used to compare the result with a neutral value of 3 for each sentence and 21 for the total (7 sentences) with significantly higher values indicating positive results. A p-value of <0.05 was used to determine the statistical significance. Statistical analysis was carried out using IBM SPSS software version 25 (SPSS Inc., Chicago, IL, USA).

## Results

The study involved 350 final-year medical students. Gender distribution was nearly balanced, with 51.4% female and 48.6% male. The mean age was 23.24 ± 0.8. Among the students, 60.9% (213) attained an average college grade ranging between 80–90%, while 34.3% (120) attained a grade below 80%, and 4.9% [17] exceeded a grade of 90%.

### Academic interest in basic medical sciences

**Fig 1:**
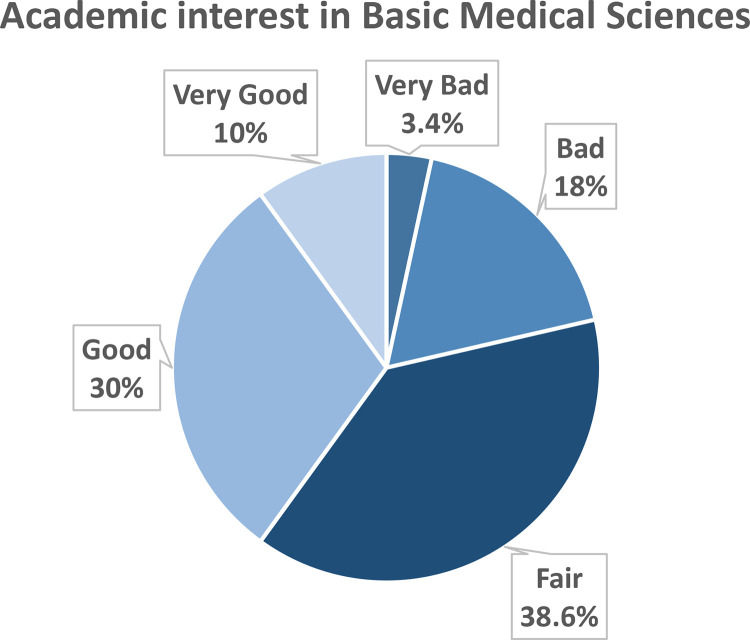
Percent of the students’ evaluations of their academic interest in basic medical sciences.

Upon asking students to evaluate the level of their academic interest in studying basic medical sciences, 40% (140) rated it as good/very good, while 21.4% (75) rated it as bad/very bad (Fig 1).

**Fig 2:**
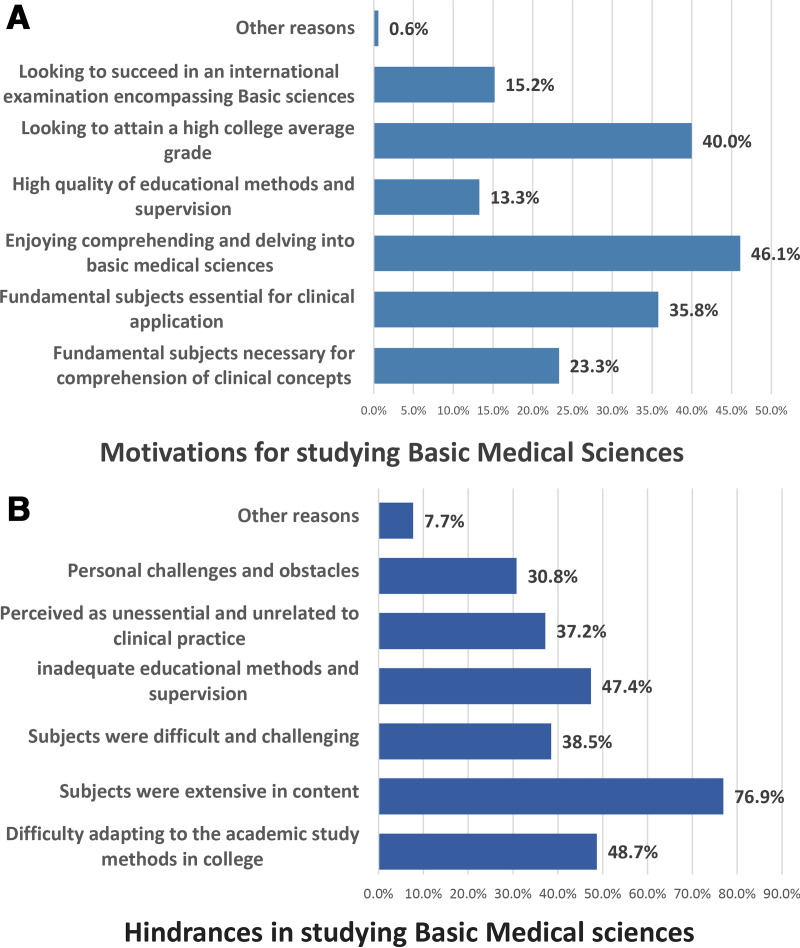
A Reasons motivate students to study Basic Medical Sciences. **B** Reasons hinder students from studying Basic Medical Sciences.

The primary motivation for studying these subjects, identified by 46.1% of students, was the enjoyment of comprehending and delving into basic sciences. Thereafter, 40% aimed for a high college grade, and 35.8% acknowledged these subjects as essential for clinical practice. Additional reasons are outlined in Fig 2-A. Conversely, most students (76.9%) cited extensive content as a major hindrance to studying these subjects, followed by 48.7% who struggled to adapt to college study methods. Other hindrances in Fig 2-B.

Students with higher average college grades exhibited an increased tendency towards focusing on basic medical sciences, as Spearman’s rank correlation coefficient demonstrated a significant positive correlation (𝜌 = 0.31, p-value < 0.001).

When diving into specific subjects, anatomy was regarded as the most focused subject (38.9%), followed by physiology (29.7%). Conversely, histology (19.4%) and embryology (18.3%) were the least focused on. Furthermore, anatomy was perceived as the easiest subject (40.9%), with physiology next (32.9%). Pharmacology (25.4%) and biochemistry (20.6%) were rated the hardest. (Table 1)

**Table 1:** Distribution of students’ choices among various subjects, n = 350.

Subject:	Most focused on	Least focused on	Easiest	Hardest
**% (n)**	**% (n)**	**% (n)**	**% (n)**
Anatomy	38.9% (136)	2.9% (10)	40.9% (143)	3.7% (13)
Physiology	29.7% (104)	0.9% (3)	32.9% (115)	0.9% (3)
Biochemistry	6.6% (23)	16% (56)	2% (7)	20.6% (72)
Pharmacology	3.1% (11)	12% (42)	2.3% (8)	25.4% (89)
Immunology	4% (14)	10.9% (38)	6.6% (23)	7.4% (26)
Pathology	2.6% (9)	10.9% (38)	2.3% (8)	8.9% (31)
Histology	4.6% (16)	19.4% (68)	2% (7)	18% (63)
Microbiology	3.7% (13)	8.9% (31)	4.9% (17)	8% (28)
Embryology	6.9% (24)	18.3% (64)	6.3% (22)	7.1% (25)

### Students’ perception of Basic Medical sciences

The overall perception regarding basic medical sciences was favorable, as evidenced by a mean score of 24.6 ± 3.6 (out of a maximum score of 35), surpassing the neutral score of 21 with statistical significance (t = 18.789, p < 0.001). A substantial proportion of students, 77.4% (271), emphasized the importance of comprehending pathophysiological mechanisms for a good physician, while 75.1% (263) valued these sciences for further medical education (table 2).

**Table 2:** Detailed statistics of statements evaluating students’ perception towards basic medical sciences.

Statement	Agreement^1^	Mean	SD	One-sample t-test	
	% (n)			t	p-value
1.Basic Science content had sufficient illustrations of clinical relevance.	44.3% (155)	3.17	0.95	3.372	0.001*
2.The content of basic medical science includes proper preparation for the clinical practice in clerkship.	20.9% (73)	2.66	0.97	−6.484	<0.001*
3.Medical basic sciences knowledge is of great importance for further medical education.	75.1% (263)	3.89	0.84	19.804	<0.001*
4.The information I have gained are essential to my future role as a physician.	59.7% (209)	3.64	0.98	12.214	<0.001*
5.Of the facets of a good physician, his/her knowledge of pathophysiological mechanisms is most important.	77.4% (271)	3.99	0.84	21.854	<0.001*
6.Applying basic medical science to clinical practice is a skill which should be reinforced early on in medical education.	74.9% (262)	3.96	0.83	21.4	<0.001*
7.It is first necessary to learn as many facts as possible in the basic sciences and then learn to apply them later on in the clinical years.	49.7% (174)	3.31	1.15	5.069	<0.001*

Responses of agree and strongly agree were combined.

*Statistically significant.

All statements showed agreement with mean scores significantly above the neutral value of 3 (p < 0.05), except for one statement on the adequacy of basic medical sciences for clinical practice, which scored significantly below 3. Detailed statistics and t-test results are in Table 2.

Additionally, a significant positive correlation was observed between students’ perceptions of basic medical sciences and their academic interest in these subjects (ρ = 0.25, p < 0.001).

### Considering Basic Medical Sciences as a prospective career

Discovering students’ willingness to pursue basic medical sciences as a prospective career revealed that only 6.6% of students [23] expressed such an intention, while a majority of 68.6% (240) declined, and 24.9% (87) were uncertain.

**Fig 3:**
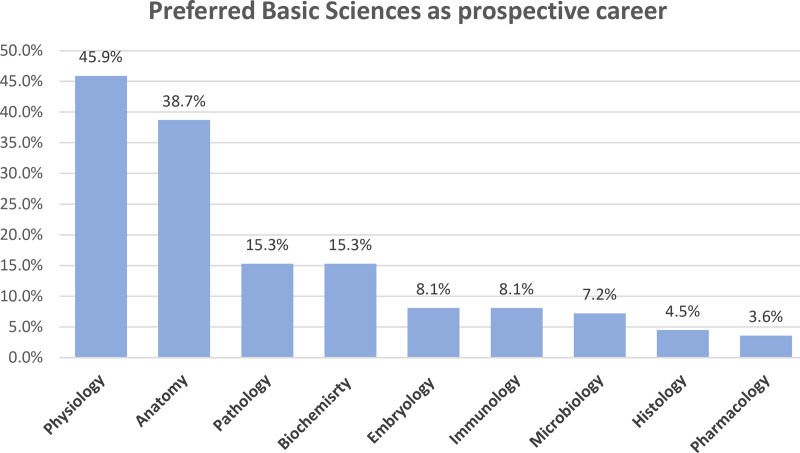
Percents of students’ preference for basic sciences as a prospective career.

**Fig 4:**
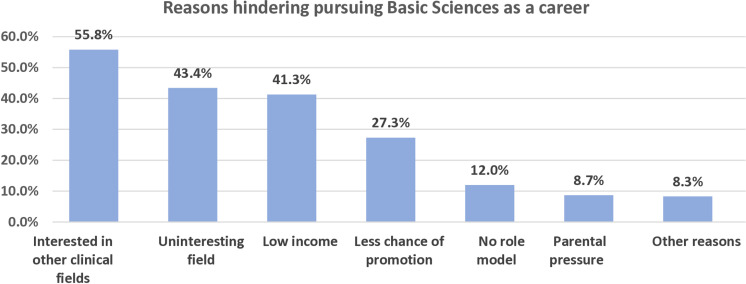
Percents of reasons that hinder students from pursuing basic medical sciences as a prospective career.

Among those interested in pursuing such a career, physiology was the most preferred, chosen by 45.9%, followed by anatomy 38.7%, then pathology and biochemistry 15.3% each. Other sciences constituted a small proportion (Fig 3). Conversely, among those who declined, reasons included a preference for clinical fields (55.8%), perceiving basic sciences as uninteresting (43.4%), and concerns regarding the potential for low-income careers (41.3%). Additional reasons are in Fig 4.

## Discussion

In our study, we aimed to explore students’ perspectives on basic medical sciences and their significance. Overall, 40% of students expressed interest in these subjects, primarily due to their desire to understand them in depth. Conversely, the main reason for students’ lack of interest was the dense content of the material. While students generally held positive views about basic medical sciences, only 6.6% were interested in pursuing them as a future profession.

Comparatively, a study from Japan reported 24.5% interest in basic medical sciences [15], while another study indicated 74% [16]. Our findings, at 40%, fall between these two extremes. Unlike previous studies that focused solely on interest percentages, our research also sought to uncover the reasons behind students’ attitudes and to assess their interest in specific subjects within basic medical sciences. The goal was to identify the easiest and most challenging subjects and recommend approaches to enhance students’ interest in these sciences.

We discovered that 46% of students were drawn to these sciences because they enjoyed comprehending and delving into them, likely influenced by the early exposure during their university education when new students tend to have scientific curiosity. Additionally, 40% expressed interest in achieving high academic grades. A notable correlation emerged between students’ average grades and their interest in basic medical sciences, suggesting that a solid grasp of these subjects enhances the understanding of disease pathophysiology, aiding exam performance.

However, many students cited the extensive content as a barrier to recalling clinically relevant information, leading to frustration and disinterest. Furthermore, nearly half reported difficulties adapting to college study methods, which differ significantly from those in high school. This transition requires self-directed learning skills that may not be fully developed in high school students. A key factor that could enhance the acceptance of basic medical sciences among medical students is the introduction of interactive education represented by social media and various medical programs, making learning such solid subjects more engaging and easier to retain [17,18].

Our study revealed that anatomy is the most focused subject among students in basic medical sciences, aligning with a previous study that identified anatomy as the most engaging subject. This popularity may stem from its interactive nature, supported by illustrations and dissection labs. However, while the aforementioned study described anatomy as content-dense, our findings suggested that students viewed it as the easiest subject [2]. This discrepancy could be attributed to variations in the curricula or the number of class sessions dedicated to anatomy.

Physiology was ranked second in focus and ease, largely because of its critical role in understanding disease mechanisms and clinical practice [19]. In contrast, histology and embryology were the least interesting subjects for students. The lack of interest in embryology may be linked to low confidence in learning the subject, highlighting the need for appropriate teaching methods [20]. Similarly, histology’s relevance in clinical practice remains overlooked. However, many underutilized basic medical sciences serve as a foundation for the rapidly evolving field of biomedicine. Advancements in cell and gene therapies highlight the need to integrate modern biotechnological approaches into the curriculum, equipping students with a deeper understanding of disease mechanisms and emerging treatments [21,22]. Similarly, incorporating molecular techniques into medical education reinforces their role in improving diagnostic accuracy and advancing personalized medicine [23,24].

Through careful examination of students’ opinions regarding basic medical sciences and their clinical correlations, over three-quarters of the students believed that knowledge of pathophysiology is essential for a competent physician, consistent with a separate study where 61% recognized the importance of physiology during clinical years [14]. This underscores the role of pathophysiology in linking disease pathological mechanisms with their clinical manifestations. As our study has shown, 75% of students valued basic medical sciences for further medical education, contrasting with another study suggesting that clinical knowledge can be gained without a foundation in basic sciences [4]. This difference may arise from variations in medical schools, curricula, and the teaching methods adopted.

Importantly, only 21% of students felt that basic medical sciences have provided appropriate practical training, echoing another study’s finding of 23% [14]. These results clearly emphasize the importance of reducing the intensive content of basic sciences and focusing on clinically relevant information.

Furthermore, our results showed that only 6.6% of students agreed to pursue basic sciences as a prospective career while the majority of students 68.6% declined. This aligns with studies in Ethiopia [2] and Lahore [3], where only 7.8% and 8.1% of students, respectively, expressed approval.

The reasons for students’ low willingness vary by country depending on the country’s educational, economic, and cultural conditions. In our study, the chief reason was students’ interest in other clinical fields (55.8%). This finding was aligned with a study conducted in Nepal which ranged students’ interests as the following: clinical surgical (50.9%), clinical medical (45.3%), and basic medical (3.9%) sciences [25]. This may be due to lower salaries compared to the clinical fields. The second most common reason in our study was low income as indicated by 41.3%. A similar situation has been observed in several countries like Ethiopia, Australia, France, and Japan, where financial constraints play an important role [2].

Additional reasons that cannot be ignored are a perception of basic sciences as uninteresting, less chance of promotion, no role model, and parental pressure. In this context, improving these subjects’ curriculum helps to increase students’ interest in this field. Parental pressure is an apparent obstacle in Syria due to misconceptions that basic sciences offer limited income and that medicine is confined to known disciplines like surgery, internal medicine, and gynecology. In addition, having a good role model motivates students to pursue this career.

Among students interested in basic sciences as a career, physiology garnered the most interest with 45.9%. Students find it interesting and essential for understanding both normal and abnormal physiological mechanisms, which is crucial for accurately comprehending diseases and aiding researchers in developing new treatments. This is the opposite of a study conducted in the United Arab Emirates showed that 40% of the final year students refused to take physiology as a future profession [26]. Anatomy was ranked second by 38.7% of students as it is an interactive and comprehensible subject with illustrations and laboratories to work in. Fewer students chose other sciences. Thus, defining and developing specializations in basic sciences will attract more participants.

### Recommendations

Based on our findings, several measures should be considered to improve students’ engagement with basic medical sciences and address the challenges they encounter:

Raising Awareness: Misconceptions about basic medical sciences should be addressed by emphasizing their fundamental role in medical education and clinical practice. This can help students recognize their long-term significance beyond academic performance.

Curriculum Enhancement: The intensity of basic science subjects should be reduced to provide essential knowledge without overwhelming students with unnecessary details. Strengthening the clinical relevance of these subjects can improve student engagement and knowledge retention.

Support for Self-Directed Learning: Structured training in self-directed learning methods, including problem-based learning (PBL), time management strategies, and interactive learning approaches, should be integrated early in medical education to facilitate a smoother transition from high school to university learning.

Career Guidance and Mentorship: Career awareness programs should be implemented to help students align their professional aspirations with their interests. These programs should highlight the significance of basic sciences in research and academia and encourage students to explore diverse opportunities in these fields.

Advancing Basic Science Specializations: Defining and promoting specialized fields within basic medical sciences, along with improving career pathways and financial incentives, could attract more medical graduates to pursue them.

### Limitations and future directions

This study was conducted at a single institution, limiting the generalizability of its findings. Future research should include multiple universities and international comparisons to assess curriculum variations and their impact on students’ perceptions.

Restricting participation to final-year students excludes perspectives from earlier-year students and graduates. Early-year students may have different attitudes, while graduates could offer insights after transitioning into clinical practice. Including diverse academic stages could provide a deeper understanding of evolving perceptions. Additionally, a longitudinal study could track changes from preclinical years to postgraduate practice, offering insights into the long-term impact of basic science education on clinical training and career choices.

Addressing these limitations will strengthen future research and contribute to optimizing medical education.

## Conclusion

This study highlighted students’ positive views on Basic Medical Sciences, noting their critical role in comprehending disease pathophysiology for medical education and effective practice while addressing their deficiency in delivering adequate practical training. These findings underscore the need to reduce the intensive content of basic medical sciences, focus on clinically relevant information, and enhance their retention through innovative practical methods that serve aspects of clinical practice.
